# Analysis of Residential Segregation and Racial and Ethnic Disparities in Severe Maternal Morbidity Before and During the COVID-19 Pandemic

**DOI:** 10.1001/jamanetworkopen.2022.37711

**Published:** 2022-10-20

**Authors:** Peiyin Hung, Jihong Liu, Chelsea Norregaard, Yiwen Shih, Chen Liang, Jiajia Zhang, Bankole Olatosi, Berry A. Campbell, Xiaoming Li

**Affiliations:** 1 Department of Health Services Policy and Management, University of South Carolina Arnold School of Public Health, Columbia; 2 South Carolina SmartState Center for Healthcare Quality, University of South Carolina Arnold School of Public Health, Columbia; 3 Department of Epidemiology and Biostatistics, University of South Carolina Arnold School of Public Health, Columbia; 4 Division of Maternal-Fetal Medicine, Department of Obstetrics and Gynecology, University of South Carolina School of Medicine, Columbia; 5 Department of Health Promotion Education and Behavior, University of South Carolina Arnold School of Public Health, Columbia

## Abstract

**Importance:**

Persistent racial and ethnic disparities in severe maternal morbidity (SMM) in the US remain a public health concern. Structural racism leaves women of color in a disadvantaged situation especially during COVID-19, leading to disproportionate pandemic afflictions among racial and ethnic minority women.

**Objective:**

To examine racial and ethnic disparities in SMM rates before and during the COVID-19 pandemic and whether the disparities varied with level of Black residential segregation.

**Design, Setting, and Participants:**

A statewide population-based retrospective cohort study used birth certificates linked to all-payer childbirth claims data in South Carolina. Participants included women who gave birth between January 2018 and June 2021. Data were analyzed from December 2021 to February 2022.

**Exposures:**

Exposures were (1) period when women gave birth, either before the pandemic (January 2018 to February 2020) or during the pandemic (March 2020 to June 2021) and (2) Black-White residential segregation (isolation index), categorizing US Census tracts in a county as low (<40%), medium (40%-59%), and high (≥60%).

**Main Outcomes and Measures:**

SMM was identified using *International Statistical Classification of Diseases, Tenth Revision, Clinical Modification (ICD-10-CM)* codes developed by the US Centers for Disease Control and Prevention. Multilevel logistic regressions with an interrupted approach were used, adjusting for maternal-level and facility-level factors, accounting for residential county-level random effects.

**Results:**

Of 166 791 women, 95 098 (57.0%) lived in low-segregated counties (mean [SD] age, 28.1 [5.7] years; 5126 [5.4%] Hispanic; 20 523 [21.6%] non-Hispanic Black; 62 690 [65.9%] White), and 23 521 (14.1%) women (mean [SD] age, 28.1 [5.8] years; 782 [3.3%] Hispanic; 12 880 [54.8%] non-Hispanic Black; 7988 [34.0%] White) lived in high-segregated areas. Prepandemic SMM rates were decreasing, followed by monthly increasing trends after March 2020. On average, living in high-segregated communities was associated with higher odds of SMM (adjusted odds ratio [aOR], 1.61; 95% CI, 1.06-2.34). Black women regardless of residential segregation had higher odds of SMM than White women (aOR, 1.47; 95% CI, 1.11-1.96 for low-segregation; 2.12; 95% CI, 1.38-3.26 for high-segregation). Hispanic women living in low-segregated communities had lower odds of SMM (aOR, 0.48; 95% CI, 0.25-0.90) but those living in high-segregated communities had nearly twice the odds of SMM (aOR, 1.91; 95% CI, 1.07-4.17) as their White counterparts.

**Conclusions and Relevance:**

Living in high-segregated Black communities in South Carolina was associated with racial and ethnic SMM disparities. During the COVID-19 pandemic, Black vs White disparities persisted with no signs of widening gaps, whereas Hispanic vs White disparities were exacerbated. Policy reforms on reducing residential segregation or combating the corresponding structural racism are warranted to help improve maternal health.

## Key Points

**Question:**

Was living in a highly segregated Black community associated with severe maternal morbidity (SMM) before and during the COVID-19 pandemic?

**Findings:**

In this cohort study of 166 791 South Carolina women with childbirths from January 2018 to June 2021, Black and Hispanic women living in high-segregated Black communities had higher odds of SMM than their counterparts living in less segregated communities. During the pandemic, Black vs White disparities in SMM persisted, while the Hispanic vs White disparities were exacerbated.

**Meaning:**

These findings suggest that policy initiatives on improving maternal health should combat the corresponding structural racism associated with residential segregation.

## Teaser

This cohort study evaluates the association between residential segregation and racial and ethnic disparities in severe maternal morbidity before and during the COVID-19 pandemic.

## Introduction

Severe maternal morbidity (SMM) rates in the US tripled from 49.5 in 1993 to 146.6 cases per 10 000 childbirths in 2014.^1^ These SMM rates are unevenly distributed geographically and socioeconomically, with the highest rate among low-income women who delivered at hospitals in Southern states.^2,3,4,5^ South Carolina’s pregnancy-related mortality rate, much like SMM rates, is among the highest in the US, with 25.5 deaths per 100 000 in 2014 to 2018.^6^ Black and women of other minority groups living in South Carolina experience a 3-fold higher pregnancy-related mortality rate (46.3 deaths per 100 000) compared with White women (13.7 deaths per 100 000).^6^ The rising SMM rate and persistent racial and ethnic disparities trigger public health concerns, not only due to the immediate burden faced by vulnerable women, but also due to potentially lasting effects on women’s health over a life course or along family lines across generations.^7^

The origin of these racial disparities in SMM is complex and multifaceted.^8^ At the micro level, maternal sociodemographic factors (eg, education and socioeconomic status), health behaviors (eg, prenatal care adequacy, smoking, diet, physical activity, and gestational weight gain), and preexisting maternal conditions (eg, hypertension, obesity, and diabetes) are associated with SMM disparities.^5,9^ At the macro level, structural racism and discrimination—historical and/or current oppression that results in disparities in access to opportunities and resources—might shape pregnant individuals’ lived experience at multiple levels,^5,10^ including community (eg, residential segregation, housing, access to healthy food, and transportation), health care organization (eg, access to high quality care and receipt of risk-appropriate treatments), and state (eg, Medicaid eligibility policy), further exacerbating racial and ethnic inequities in maternal and birth outcomes in the US.^10,11,12^ Despite several sociological theories trying to explain the multilevel pathways of structural racism on racial and ethnic health inequities,^13,14,15^ limited evidence has categorized their role in SMM, especially during the COVID-19 pandemic.

The COVID-19 pandemic has led to unprecedented societal disruption with a wide range of social and systemic influence at multiple levels. Social isolation, financial distress, community crowdedness, food insecurity, neighborhood violence, technology literacy, and access to perinatal care are among key structural factors that are associated with racial and ethnic disparities in both physical and mental health outcomes.^16,17,18,19^ The pandemic has affected communities of color the hardest^20,21,22^; Black and Hispanic pregnant women appear to have disproportionate SARS-CoV-2 exposure, infection, and death rates.^23,24,25^ Structural racism, often measured by residential segregation, is compounding the COVID-19 pandemic crisis, potentially leaving women of color in a disadvantaged situation in terms of psychosocial stress, employment, education, digital access, and income.^17,20,21,22^ Understanding how structural racism is associated with SMM disparities, especially amid the COVID-19 pandemic, is essential to identify opportunities to eliminate racial and ethnic disparities in SMM and develop tailored interventions for corresponding risk factors, thus improving maternal health equity.

Few definitive data have concluded how the COVID-19 pandemic has changed the incidences of SMM across maternal racial and ethnic groups, especially through racial residential segregation and its corresponding socioeconomic deprivation. We used a statewide population-based database and the months that have elapsed since the COVID-19 pandemic as a natural experiment to assess its association with SMM rates by racial residential segregation.

## Methods

### Data Sources

This statewide cohort study used data of all-payer childbirth records in South Carolina from inpatient discharges, emergency department visits, outpatient surgery, and other outpatient services, linked to birth certificates for maternal demographic, parity, chronic disease risk factors, and prenatal care utilization patterns. The South Carolina Office of Revenue and Fiscal Affairs collated databases and provided the authors with a deidentified linked database with unique identifiers for every individual. The study was considered exempt from the need for informed consent and institutional review board approval at the University of South Carolina due to the nature of the secondary analysis of deidentified data. The study followed the Strengthening the Reporting of Observational Studies in Epidemiology (STROBE) reporting guideline for cohort studies.

We identified all childbirth deliveries to women living in South Carolina regardless of the place of birth that occurred between January 2018 and June 2021 using *International Statistical Classification of Diseases, Tenth Revision, Clinical Modification (ICD-10-CM)* diagnoses and procedure codes, Diagnosis-Related Group codes, and *Current Procedural Terminology* codes (eTable 1 in the Supplement). The 2015 to 2019 American Community Survey (ACS) US Census tract-level racial and ethnic compositions were derived to calculate county-level residential segregation measure.

### Measures

SMM was the primary outcome and SMM without blood transfusion was the secondary outcome, defined as whether a woman experienced any of the 21 indicators developed by the Centers for Disease Control and Prevention from a childbirth hospitalization admission date to 60 days post partum.^26^

Key exposures include (1) a pandemic indicator (ie, prepandemic [January 2018 to February 2020] and peripandemic [March 2020 to June 2021] periods of childbirth delivery), and (2) a county-level residential segregation measure (isolation index) that represents the probability that Black residents would interact with White residents and vice versa across US Census tracts in a maternal residential county, categorized into low (<40%), medium (40%-59%), and high (≥60%).^27^ This isolation index was calculated across US Census tracts using non-Hispanic Black and White as population groups. Higher scores of Black vs White isolation index indicate more extensive isolation of Black residents across US Census tracts in the county, and lower scores suggest less isolation or more Black vs White exposure, compared with the average interracial exposure of the county.^11,27,28^

Covariates included maternal age at childbirth, maternal race and ethnicity (Hispanic, non-Hispanic Black, non-Hispanic White, and non-Hispanic other races, which includes Asian, American Indian, Native Hawaiian and other Pacific Islander, and multiracial groups); maternal education attainment; primary payer; prepregnancy body mass index; enrollment in Women, Infants and Children (WIC) Nutrition Program; gestational trimester of prenatal care initiation; smoking during pregnancy; previous cesarean delivery; preterm labor; plurality; urban vs rural location of health care facility; level of perinatal care; and chronic conditions (preexisting diabetes, gestational diabetes, preexisting hypertension, and pregnancy-induced hypertension). Race and ethnicity data were derived from birth certificate data in which maternal race was categorized as Black, other (Asian, American Indian, Native Hawaiian and other Pacific Islander, and multiracial groups), and White, while Hispanic was a separate variable. We grouped childbirth records with missing values into a separate stratum for each variable.

### Statistical Analysis

We first used frequencies and percentages to describe maternal sociodemographic characteristics before the pandemic and during the pandemic across Black vs White residential segregation categories and across maternal race and ethnicity groups in each residential segregation level. Differences were compared using Pearson χ^2^ tests for nominal variables and Mantel-Haenszel tests of trend for ordinal variables. Next, trends in SMM rates per race and ethnicity group in a segregation level were assessed using the Mann-Kandall tests. Furthermore, multilevel mixed-effects logistic regressions were used, adjusting for maternal sociodemographic, clinical, and behavioral factors; facility-level perinatal care level; rurality for place of birth; and accounting for maternal residential county-level random effects and interrelated monthly trends. In the modeling procedure, we performed an interrupted time series analysis by including a term for prepandemic monthly trend, a pandemic variable to indicate peripandemic childbirth, and a term to estimate changes in monthly trends during the peripandemic period to test the association between the COVID-19 pandemic and SMM. We also conducted a set of sensitivity analyses to look at 2-way interactions of prepandemic and peripandemic monthly trends with segregation and race and ethnicity. The racial and ethnic disparities, within each level of segregation, and pandemic-associated changes were assessed through odds ratios (OR) and 95% CIs. All statistical analyses were undertaken using Stata statistical software version 16 (StataCorp), with statistical significance set at *P* < .05 (2-tailed). Data were analyzed from December 2021 to February 2022.

## Results

### Sociodemographic Characteristics by Residential Segregation

Of 166 791 childbirths, 95 098 (57.0%) were to women in low-segregated Black communities pre–COVID-19 (mean [SD] age, 28.1 [5.7] years; 20 523 [21.6%] Black, 5126 [5.4%] Hispanic, 62 690 [65.9%] White), 48 172 (28.9%) to women in medium-segregated communities (mean [SD] age, 28.1 [5.8] years; 17 863 [37.1%] Black, 1899 [3.9%] Hispanic, 25 129 [52.2%] White), and 23 521 (14.1%) to women in high-segregated communities (12 880 [54.8%] Black, 782 [3.3%] Hispanic, 7988 [34.0%] White) (Table). Compared with those in low-segregated areas, women in high-segregated communities were more likely to have Medicaid or other public insurance (vs private), to be diagnosed with obesity before pregnancy, to enroll in the WIC program, to have inadequate prenatal care visits, to have preexisting hypertension, and to deliver in rural health care facilities or hospitals with level II perinatal care (vs level III).

**Table.** Maternal and Facility Characteristics of Women Giving Birth in South Carolina Before (January 2018 to Febraury 2020) and During (March 2020 to June 2021) the COVID-19 Pandemic by Black-White Residential Segregation Index

Characteristic	Childbirths, No. (%)							
	Low segregation <40% (n = 95 098)		Medium segregation 40%-59% (n = 48 172)		High segregation ≥60% (n = 23 521)		*P* values, high vs low^a^	
	Pre–COVID-19 (n = 58 840)	Peri–COVID-19 (n = 36 258)	Pre–COVID-19 (n = 29 924)	Peri–COVID-19 (n = 18 248)	Pre–COVID-19 (n = 14 651)	Peri–COVID-19 (n = 8870)	Pre–COVID-19	Peri–COVID-19
Maternal age, y								
<15	32 (0.1)	15 (0.04)	25 (0.1)	15 (0.1)	10 (0.1)	17 (0.2)	.09	.36
15-19	3664 (6.2)	1970 (5.4)	1989 (6.7)	1156 (6.3)	936 (6.4)	528 (6.0)		
20-24	13 388 (22.8)	7784 (21.5)	6887 (23.0)	4059 (22.2)	3331 (22.7)	1991 (22.5)		
25-29	18 433 (31.3)	11 322 (31.2)	8993 (30.1)	5246 (28.8)	4492 (30.7)	2661 (30.0)		
30-34	15 113 (25.7)	9821 (27.1)	7600 (25.4)	4929 (27.0)	3753 (25.6)	2337 (26.4)		
≥35	8210 (14.0)	5346 (14.7)	4430 (14.8)	2843 (15.6)	2129 (14.5)	1336 (15.1)		
Maternal race and ethnicity								
Hispanic	3216 (5.5)	1910 (5.3)	1116 (3.7)	783 (4.3)	486 (3.3)	296 (3.3)	<.001	<.001
Non-Hispanic								
Black	12 666 (21.5)	7857 (21.7)	11 171 (37.3)	6692 (36.7)	8007 (54.7)	4873 (54.9)		
White	38 842 (66.0)	23 848 (65.8)	15 568 (52.0)	9561 (52.4)	4997 (34.1)	2991 (33.7)		
Others^b^	4116 (7.0)	2643 (7.3)	2069 (6.9)	1212 (6.6)	1161 (7.9)	710 (8.0)		
Maternal education								
No high school diploma	8014 (13.6)	4432 (12.2)	3999 (13.4)	2304 (12.6)	1612 (11.0)	908 (10.2)	.15	<.001
High school diploma	14 556 (24.7)	9275 (25.6)	7617 (25.5)	4822 (26.4)	3717 (25.4)	2407 (27.1)		
Some college	19 616 (33.3)	11 736 (32.4)	9796 (32.7)	5680 (31.1)	5051 (34.5)	3010 (33.9)		
Bachelor’s degree	10 772 (18.3)	6887 (19.0)	5375 (18.0)	3396 (18.6)	2540 (17.3)	1533 (17.3)		
Graduate school	5709 (9.7)	3809 (10.5)	3009 (10.1)	2002 (11.0)	1658 (11.3)	990 (11.2)		
Unknown	173 (0.3)	119 (0.3)	128 (0.4)	44 (0.2)	73 (0.5)	22 (0.3)		
Primary payer								
Private	23 990 (40.8)	15 049 (41.5)	11 379 (38.0)	7032 (38.5)	5031 (34.3)	3034 (34.2)	<.001	<.001
Medicaid	25 467 (43.3)	14 803 (40.8)	13 436 (44.9)	7609 (41.7)	6501 (44.4)	3868 (43.6)		
Other public	6969 (11.8)	3886 (10.7)	4380 (14.6)	2848 (15.6)	2806 (19.2)	1566 (17.7)		
Uninsured	1595 (2.7)	924 (2.6)	334 (1.1)	182 (1.0)	155 (1.1)	111 (1.3)		
Unknown	819 (1.4)	1596 (4.4)	395 (1.3)	577 (3.2)	158 (1.1)	291 (3.3)		
Prepregnancy body mass index^c^								
Underweight	904 (1.5)	429 (1.2)	311 (1.0)	309 (1.7)	113 (0.8)	41 (0.5)	<.001	<.001
Normal	22 045 (37.5)	12 964 (35.8)	11 608 (38.8)	6697 (36.7)	4754 (32.5)	2723 (30.7)		
Overweight	14 873 (25.3)	9501 (26.2)	7299 (24.4)	4543 (24.9)	3652 (24.9)	2271 (25.6)		
Obesity	19 087 (32.4)	12 357 (34.1)	9734 (32.5)	6176 (33.8)	5699 (38.9)	3581 (40.4)		
Unknown	1931 (3.3)	1007 (2.8)	972 (3.3)	523 (2.9)	433 (3.0)	254 (2.9)		
Women, Infants and Children program								
Yes	19 546 (33.2)	10 236 (28.2)	12 618 (42.2)	6743 (37.0)	6705 (45.8)	3607 (40.7)	<.001	<.001
No	38 266 (65.0)	25 580 (70.6)	16 774 (56.1)	11 272 (61.8)	7748 (52.9)	5204 (58.7)		
Unknown	1028 (1.7)	442 (1.2)	532 (1.8)	233 (1.3)	198 (1.4)	59 (0.7)		
Prenatal care initiation								
First trimester	45 292 (77.0)	28 473 (78.5)	22 285 (74.5)	13 847 (75.9)	10 684 (72.9)	6324 (71.3)	<.001	<.001
Second trimester	10 612 (18.0)	6016 (16.6)	5960 (19.9)	3398 (18.6)	2964 (20.2)	1675 (18.9)		
Third trimester or none	2936 (5.0)	1769 (4.9)	1679 (5.6)	1003 (5.5)	1003 (6.9)	871 (9.8)		
Tobacco use during pregnancy								
Yes	5323 (9.1)	2642 (7.3)	2172 (7.3)	1053 (5.8)	626 (4.3)	295 (3.3)	<.001	<.001
No	53 490 (90.9)	33 598 (92.7)	27 703 (92.6)	17 185 (94.2)	13 996 (95.5)	8573 (96.7)		
Unknown	27 (0.1)	18 (0.1)	49 (0.2)	10 (0.1)	29 (0.2)	2 (<0.01)		
Previous cesarean delivery								
Yes	9741 (16.6)	6124 (16.9)	4789 (16.0)	2926 (16.0)	2514 (17.2)	1394 (15.7)	.22	.012
No	49 099 (83.4)	30 134 (83.1)	25 135 (84.0)	15 322 (84.0)	12 137 (82.8)	7476 (84.3)		
Preexisting diabetes								
Yes	554 (0.9)	467 (1.3)	371 (1.2)	233 (1.3)	172 (1.2)	117 (1.3)	<.001	.57
No	58 286 (99.1)	35 791 (98.7)	29 553 (98.8)	18 015 (98.7)	14 479 (98.8)	8753 (98.7)		
Gestational diabetes								
Yes	3982 (6.8)	2938 (8.1)	1799 (6.0)	1282 (7.0)	821 (5.6)	578 (6.5)	<.001	<.001
No	54 858 (93.2)	33 320 (91.9)	28 125 (94.0)	16 966 (93.0)	13 830 (94.4)	8292 (93.5)		
Preexisting hypertension								
Yes	1868 (3.2)	1540 (4.3)	1112 (3.7)	701 (3.8)	603 (4.1)	379 (4.3)	<.001	.54
No	56 972 (96.8)	34 718 (95.8)	28 812 (96.3)	17 547 (96.2)	14 048 (95.9)	8491 (95.7)		
Pregnancy-induced hypertension								
Yes	5687 (9.7)	3630 (10.0)	2117 (7.1)	1516 (8.3)	997 (6.8)	530 (6.0)	<.001	.32
No	53 153 (90.3)	32 628 (90.0)	27 807 (92.9)	16 732 (91.7)	13 654 (93.2)	8340 (94.0)		
Preterm labor								
Yes	5775 (9.8)	3865 (10.7)	3081 (10.3)	1973 (10.8)	1685 (11.5)	1007 (11.4)	.002	.28
No	53 065 (90.2)	32 393 (89.3)	26 843 (89.7)	16 275 (89.2)	12 966 (88.5)	7863 (88.7)		
Plurality								
Singleton	57 850 (98.3)	35 625 (98.3)	29 433 (98.4)	17 922 (98.2)	14 385 (98.2)	8711 (98.2)	.62	.89
Multiple	990 (1.7)	633 (1.8)	491 (1.6)	326 (1.8)	266 (1.8)	159 (1.8)		
Urban vs rural location of facility								
Rural	4086 (6.9)	2477 (6.8)	10 003 (33.4)	5766 (31.6)	2429 (16.6)	1274 (14.4)	<.001	<.001
Urban	53 947 (91.7)	32 186 (88.8)	19 540 (65.3)	11 908 (65.3)	12 075 (82.4)	7305 (82.4)		
Unknown	807 (1.4)	1595 (4.4)	381 (1.3)	574 (3.2)	147 (1.0)	291 (3.3)		
Level of perinatal care								
Level I	7885 (13.4)	5765 (15.8)	4669 (15.6)	2464 (13.5)	512 (3.5)	372 (4.1)	<.001	<.001
Level II	18 064 (30.7)	11 059 (30.5)	14 094 (47.1)	8850 (48.5)	6139 (41.9)	4196 (47.3)		
Level III	23 006 (39.1)	13 887 (38.3)	9366 (31.3)	5584 (30.6)	7575 (51.7)	3858 (43.5)		
Unknown	9885 (16.8)	5547 (15.3)	1795 (6.0)	1350 (7.4)	425 (2.9)	444 (5.0)		

^a^ *P* values were calculated using Mantel-Haenszel tests of trend for ordinal variables and χ^2^ tests for nominal variables.

^b^ Other race groups include Asian, American Indian, Native Hawaiian and other Pacific Islander, and multiracial groups.

^c^ Body mass index is calculated as weight in kilograms divided by height in meters squared.

Compared with those in low-segregated communities, women of all race and ethnicity groups living in high-segregated communities were more likely to have childbirths at age 30 years or older, to have obesity before pregnancy, and to enroll in WIC programs (except non-Hispanic White women). In addition to these differences, non-Hispanic women across all races in high-segregated communities had higher educational attainment. Unlike non-Hispanic women, Hispanic women living in high-segregated areas were more likely to have no high school diploma, to be primarily Medicaid insured or have no health insurance, and have preexisting diabetes than Hispanic women in low-segregated areas (eTable 2 in the Supplement).

### SMM by Residential Segregation Across Race and Ethnicity

**Figure 1.**
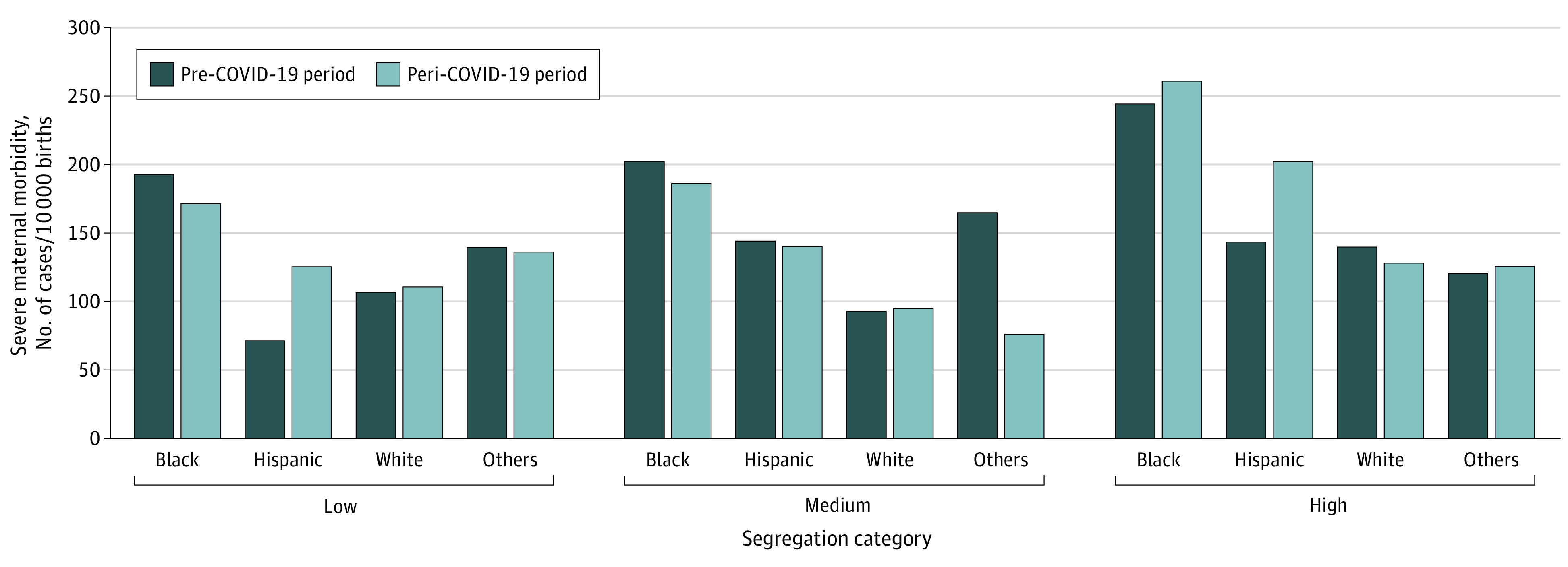
Unadjusted Rates of Severe Maternal Morbidity by Level of Black-White Residential Segregation, Before (January 2018 to February 2020) and During (March 2020 to June 2021) the COVID-19 Pandemic Residential segregation measure (isolation index) represents the probability that Black residents would interact with White residents and vice versa across US census tracts in a maternal residential county, categorized into low (<40%), medium (40%-59%), and high (≥60%).

Unadjusted rates of SMM measures were highest among mothers who reside in high-segregated areas (Figure 1). On average, living in high-segregated communities was associated with higher odds of SMM (adjusted OR [aOR], 1.61; 95% CI, 1.06-2.34). SMM rates among Black women were highest overall, in both prepandemic and peripandemic periods. These rates were much higher among Black women in high-segregated communities (196 [244.8 cases per 10 000] in the prepandemic period and 127 [260.6 cases per 10 000] in the peripandemic period) than Black women living in low-segregated communities (244 [192.6 cases per 10 000] in the prepandemic period and 135 [171.8 cases per 10 000] in the peripandemic period). Of those in high-segregated communities, Hispanic mothers had a substantially higher SMM rate (202.7 cases per 10 000) during the COVID-19 pandemic period compared with prepandemic (144.9 cases per 10 000). SMM rates among Hispanic women in high-segregated areas were also higher than Hispanic women in low-segregated areas and non-Hispanic white women in both high and low-segregated communities. Similar trends in SMM without blood transfusion exist (eFigure in the Supplement).

**Figure 2.**
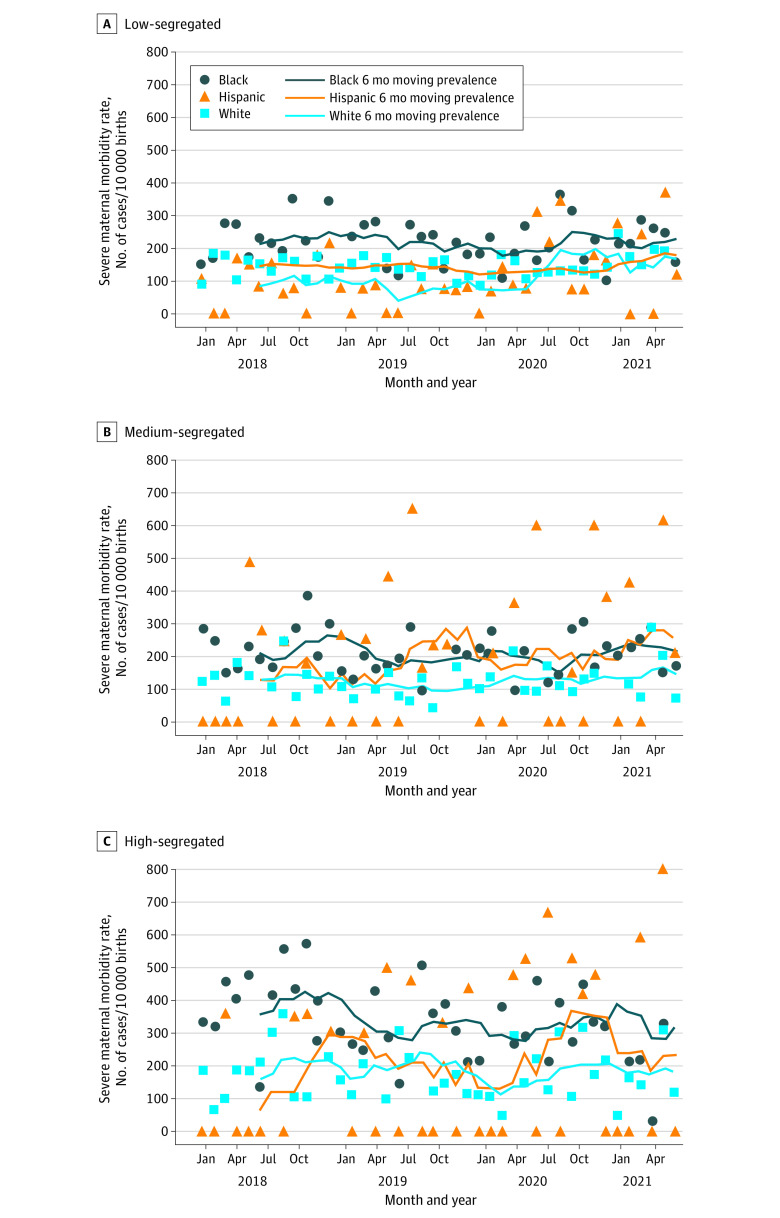
Monthly and 6-Month Moving Prevalence of Severe Maternal Morbidity Among White, Black, and Hispanic Women Giving Birth in January 2018 to June 2021 by County-Level Black-White Residential Segregation Level Residential segregation measure (isolation index) represents the probability that Black residents would interact with White residents and vice versa across US Census tracts in a maternal residential county, categorized into low (<40%), medium (40%-59%), and high (≥60%). Data for non-Hispanic other race group and some data for Hispanic group are suppressed due to less than 10 cases. All race and ethnicity groups’ rates were calculated across all childbirths of non-Hispanic White (White), non-Hispanic Black (Black), Hispanic, and other race populations.

During January 2018 to June 2021, Black women regardless of residential segregation had consistently higher SMM rates than their White counterparts (Figure 2). Black women in high-segregated communities had a slightly decreasing trend in January 2019 to March 2020, but this decreasing trend was reversed in April 2020. SMM rates for Hispanic women in low-segregated communities had a continual increasing trend (Kendall τ = 0.21; *P* = .02) but rates for those in high-segregated communities fluctuated.

**Figure 3.**
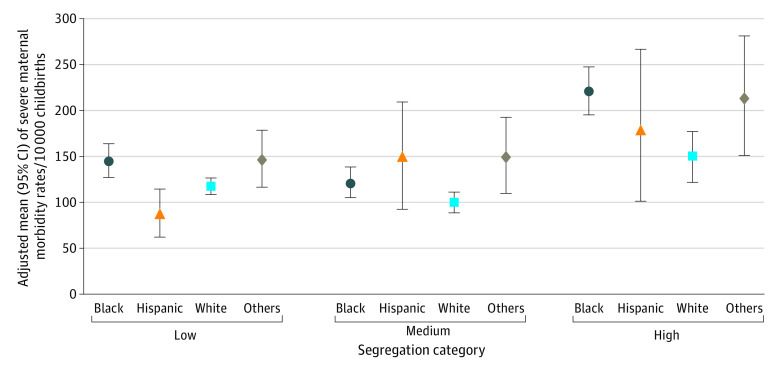
Adjusted Severe Maternal Morbidity Rates per 10 000 Childbirths by Maternal Residential Segregation and Race and Ethnicity Adjusted means and 95% CIs (upper and lower) of severe maternal morbidity rates were calculated from model estimates presented in eTable 3 in the Supplement and average covariate values in the Table.

Black and Hispanic women living in high-segregated Black communities had higher probabilities of SMM than their counterparts living in low-segregated communities (Figure 3). Black vs White disparities in SMM persisted across communities by segregation, but disparities increased with segregation level (low-segregated: aOR, 1.47; 95% CI, 1.11-1.96; high-segregated: aOR, 2.12; 95% CI, 1.38-3.26) (eTable 3 in the Supplement). Hispanic women living in low-segregated communities had lower odds of SMM (aOR, 0.48; 95% CI, 0.25-0.91) but those living in high-segregated communities had nearly twice the odds of SMM (aOR, 1.90; 95% CI, 1.07-4.17) as their White counterparts. On average, the odds of SMM were decreasing before March 2020 (prepandemic monthly trends: aOR, 0.98; 95% CI, 0.97-1.00), with no significant immediate level changes in March 2020 (aOR, 1.19; 95% CI, 0.85-1.67), but followed by monthly increasing trends after March 2020 (aOR, 1.05; 95% CI, 1.02-1.08). Pandemic changes in SMM did not vary with Black residential segregation but varied with maternal race and ethnicity. In the sensitivity analysis where 3-way interactions across pandemic indicator, race and ethnicity, and isolation index level were included, peripandemic increasing trends were not significantly different between Black and White women, but were higher among Hispanic women (aOR, 1.48; 95% CI, 1.14-2.28) vs their White counterparts.

## Discussion

In this statewide population-based cohort study, we found that living in high-segregated Black communities was associated with greater risk of maternal morbidity, independent of individual demographic, clinical, and economic conditions. In low-segregated communities, the odds of SMM among non-Hispanic Black women were 1.47 times higher than the odds for White women, and in high-segregated communities, this Black vs White disparity was exacerbated. Interestingly, Hispanic women residing in the low-segregated communities had lower odds of SMM than non-Hispanic White women, but Hispanic women living in high-segregated Black communities had nearly 2-fold the odds of SMM compared with non-Hispanic White women. This Hispanic vs White gap in high-segregated communities was particularly elevated during the pandemic. From January 2018 to February 2020, the prepandemic period, the incidence of SMM had a monthly decreasing trend, followed by an increasing monthly trend during the peripandemic period. These pandemic-associated increases in SMM persisted across levels of Black residential segregation.

In each race and ethnicity group, SMM risk was higher among minoritized residents living in high-segregated communities compared with their counterparts in low-segregated communities. This finding is consistent with existing evidence on the increased maternal health issues for women living in majority-Black neighborhoods.^29,30^ The fact that such residential segregation was associated with SMM after adjusting for individual-level variations in education, insurance, obesity, nutrition, prenatal care, smoking during pregnancy, and clinical factors has critical implications. First, the residents living in Black segregated communities had worse maternal morbidity outcomes, which might be a totality of historical and structural racism, rather than individual socioeconomic burdens. Cumulative and mutually reinforcing discrimination of housing, education, employment, criminal justice, economic opportunities, and health care put these racial minority populations at higher risk of adverse maternal outcomes. Second, during the pandemic, Black communities appear to have disproportionate COVID-19 outcomes. Furthermore, pregnant women of color who were infected with SARS-CoV-2 were much more likely to be hospitalized or die.^31,32,33^ Third, Black and Hispanic women living in the most segregated areas had less access to high-quality obstetric care than White women,^34^ likely due to the uneven or unequal distribution of high-quality hospital obstetric units.^35^ Multilevel factors such as racial residential segregation across communities, hospitals with high SMM rates, and areas with hospital obstetric closures call into question whether underlying structural racism is hindering Black women from accessing high-quality hospitals^36,37^ and, in turn, increasing their risk of SMM.^36^ To advance maternal health equity and improve population health, addressing the challenges faced in high Black segregated communities is important. For example, an inclusion of pregnant persons’ residence information and social determinant of health *ICD-10-CM* codes in the electronic health record or medical records may be an important starting point for this effort to better inform clinical prognosis planning for COVID-19 and perinatal care.

Research on the association between residential segregation or structural racism and maternal health has focused on racial disparities between Black women and White women.^30,38,39^ Our findings suggest that such Black residential segregation is not solely associated with Black vs White maternal health disparities. In the current study, the highest SMM incidence of Hispanic women was detected among those residing in high-segregated Black communities during the COVID-19 pandemic, where 200 or more of 10 000 women experienced SMM. Although Hispanic women across all levels of segregations experienced higher SMM incidences peripandemic vs prepandemic, the SMM incidence increased disproportionately in the high-segregated communities. The reasons for Black residential segregation being significantly associated with SMM risks among Hispanic populations are likely multifactorial. Higher prevalence of SARS-CoV-2 infections among Black and Hispanic persons may play a role in the increased rates of SMM in these populations.^4,31,32,33^ Also, two-thirds of Hispanic women with childbirths in South Carolina were immigrants^40^ whose inadequate prenatal care and adverse birth outcomes were documented to be associated with criminalizing immigrant policies.^41,42^ In South Carolina, only lawfully residing pregnant women would be provided with medical coverage, whereas about 20 other states provide prenatal care regardless of pregnant person’s immigration status.^43^ Additionally, these Hispanic populations are more likely to work in essential industries and the associated psychosocial stress might further increase their risks of SMM.^44,45^ Importantly, members of Hispanic populations face poorer access to health care and economic instability than non-Hispanic populations. In the current study, Hispanic women living in highly Black segregated communities had disproportionately high uninsured or Medicaid insurance rates, as well as much lower educational attainment than their Hispanic counterparts in the least segregated communities. Poverty, unstable housing, lack of transportation, and poor access to quality education, among other social determinants of health, are more common overall in Hispanic populations.^46^ Also, lack of health insurance and low health literacy, especially under the circumstance when one’s primary language was other than English, might add another layer of the difficulties faced by these Hispanic families.^46^ Together, these factors may intensify disparities in health outcomes, including SMM. This analysis provides empirical data at the intersection between race, ethnicity and structural racism in maternal health outcomes among SC women and should inform efforts to advance maternal health equity in underprivileged communities.

Ongoing efforts have called for addressing the maternal health crisis in the US.^49^ The Healthy People 2030 goals seek to reduce severe maternal complication to 0.62% and reduce maternal deaths to 15.7 per 100 000 live births.^50^ This study suggests that ways to prevent future SMM cases should pay particular attention to families living in high Black segregation communities. The intersection of SMM risks based on both race and ethnicity–based and place-based inequities requires comprehensive, inclusive, and feasible practices to address the social factors that contribute to the persistent and sadly increasing SMM rates during the pandemic. Yet, it is critical to recognize that Black and Hispanic populations are heterogeneous, and this study identified profound disadvantages among those residing in high Black segregated counties. Improving maternal outcomes for minority populations requires engagement with these families who might have distinct barriers to receive optimal health care before, during, and following childbirth.

### Limitations

Although this study highlights residential segregation as a factor associated with risk for SMMs,^47^ the results of this study should be interpreted cautiously in light of data limitations. We derived data from South Carolina population-based health utilization data of all childbirths from January 2018 to June 2021; SMM may occur up to 1 year after childbirth and this study adopted a previously documented approach by identifying SMM from childbirth admission through 2 months post partum, which should capture the majority of the incidences.^48^ Moreover, circumstances unique to South Carolina such as social structures, non-Medicaid expansion, health coverage of immigrant populations, and all-statewide counties designated whole or in part as medically underserved areas might have contributed to the findings. We examined isolation index as a measure of residential segregation because it was previously documented to be a sensitive segregation measure to perinatal health,^11,28^ but structural racism might also manifest in other forms such as institutional racism at the delivery hospitals. Additionally, due to sample size, other racial populations such as American Indian and Alaska Natives were not able to be separately studied in the current study, which warrants future investigations given their high exposure and deaths related to SARS-CoV-2.

## Conclusions

In this statewide cohort study of more than 100 000 South Carolina women with childbirths from January 2018 to June 2021, Black and Hispanic women living in high-segregated Black communities had higher odds of SMM than their counterparts living in less segregated communities, respectively. During the pandemic, Black vs White disparities in SMM persisted while the Hispanic vs White disparities were exacerbated. Policy reforms to reduce residential segregation or combat the corresponding structural racism are warranted to help improve maternal health.

## Notes

Supplement. **eTable 1. **Study Sample Inclusion and Exclusion Criteria **eTable 2. **Maternal Characteristics by Race and Ethnicity and Residential County Black Residential Segregation **eTable 3. **The COVID-19 Pandemic Changes in Severe Maternal Morbidity and Its Racial and Ethnic Disparities by Racial Residential Segregation **eFigure. **Monthly and 6-Month Moving Prevalence of Severe Maternal Morbidity Without Blood Transfusion Among White, Black and Hispanic Women Giving Birth in January 2018 to June 2021 by County-Level Black-White Residential Segregation Level
