# Supplementary material for: Analysis of Residential Segregation and Racial and Ethnic Disparities in Severe Maternal Morbidity Before and During the COVID-19 Pandemic
Source: JAMA Netw Open. 2022 Oct 20;5(10):e2237711. doi: 10.1001/jamanetworkopen.2022.37711 (PMC9585430; doi:10.1001/jamanetworkopen.2022.37711)
Supplement: Supplement. — eTable 1. Study Sample Inclusion and Exclusion Criteria eTable 2. Maternal Characteristics by Race and Ethnicity and Residential County Black Residential Segregation eTable 3. The COVID-19 Pandemic Changes in Severe Maternal Morbidity and Its Racial and Ethnic Disparities by Racial Residential Segregation eFigure. Monthly and 6-Month Moving Prevalence of Severe Maternal Morbidity Without Blood Transfusion Among White, Black and Hispanic Women Giving Birth in January 2018 to June 2021 by County-Level Black-White Residential Segregation Level [file jamanetwopen-e2237711-s001.pdf]

Supplemental Online Content

Hung P, Liu J, Norregaard C, et al. Analysis of residential segregation and racial and ethnic disparities in severe maternal morbidity before and during the COVID-19 pandemic. *JAMA Netw Open*. 2022;5(10):e2237711. doi:10.1001/jamanetworkopen.2022.37711

eTable 1. Study Sample Inclusion and Exclusion Criteria
eTable 2. Maternal Characteristics by Race and Ethnicity and Residential County Black Residential Segregation
eTable 3. The COVID-19 Pandemic Changes in Severe Maternal Morbidity and Its Racial and Ethnic Disparities by Racial Residential Segregation
eFigure. Monthly and 6-Month Moving Prevalence of Severe Maternal Morbidity Without Blood Transfusion Among White, Black and Hispanic Women Giving Birth in January 2018 to June 2021 by County-Level Black-White Residential Segregation Level

This supplemental material has been provided by the authors to give readers additional information about their work.

© 2022 Hung P et al. *JAMA Network Open.*

eTable 1. Study Sample Inclusion and Exclusion Criteria

	Inclusion for Childbirth Delivery	Exclusion for Termination of Pregnancy[a]
ICD-10-CM diagnosis codes	Z37.xx, O80, O82, O75.82	O00.x, O01.x, O02.x, O03.x, O04.x, O07.x, O08.x, Z37.1, Z37.3, Z37.4, Z37.7, O31-O31.04
ICD-10-CM procedure codes	10D07Z3-10D07Z8, 10E0XZZ, 10D00Z0-10D00Z2	10A00ZZ, 10A03ZZ, 10A04ZZ, 10A07Z6, 10A07ZW, 10A07ZX, 10A07ZZ, 10A08ZZ
DRG codes	765-768, 774-775, 783-788, 796-798, 805-807	
CPT codes	59400, 59409, 59410, 59610, 59612, 59614, 59514, 59620	59840, 59841, 59850-59852, 59855-59857

Notes: a. termination of pregnancy includes abortion of products of conception, ectopic pregnancy, and pregnancy with abortive outcome. ICD-10-CM= international classification of diseases tenth revision clinical modification; DRG=Diagnosis Related Group; CPT=Current Procedural Terminology (CPT)/Healthcare Common Procedure Coding System (HCPCS) Codes.

© 2022 Hung P et al. *JAMA Network Open.*

eTable 2. Maternal Characteristics by Race and Ethnicity and Residential County Black Residential Segregation

	Low Segregation				Medium Segregation				High Segregation			
	Non-Hispanic (NH) White	NH Black	Hispanic	NH Other	NH White	NH Black	Hispanic	NH Other	NH White	NH Black	Hispanic	NH Other
**Total Number of Childbirths**	62,690	20,523	5,126	6,759	25,129	17,863	1,899	3,281	7,988	12,880	782	1,871
	**Proportion of Childbirths**											
**Maternal Age**												
<15	0.0	0.1	0.2	0.1	0.0	0.1	0.1	0.2	0.0	0.2	0.1	0.1
15-19	5.0	8.1	8.7	6.1	4.3	9.2	10.1	7.4	3.6	7.9	7.1	4.2
20-24	21.0	27.4	23.7	18.6	18.7	28.5	27.1	20.8	15.8	26.4	21.5	18.9
25-29	31.9	31.3	26.5	28.2	28.5	31.7	26.2	26.8	30.5	30.6	27.2	29.8
30-34	27.9	21.2	22.8	27.6	30.7	19.7	21.6	26.3	33.1	21.8	27.7	28.2
35+	14.2	11.9	18.1	19.4	17.9	10.8	14.9	18.5	17.0	13.1	16.4	18.9
**Maternal Education**												
Unknown	0.2	0.4	0.7	0.5	0.2	0.5	0.6	0.8	0.3	0.4	0.3	0.9
No High School Diploma	10.6	12.1	41.8	20.1	8.4	13.9	44.1	27.9	7.2	10.9	45.7	10.4
High School Diploma	21.8	34.6	30.3	24.3	17.5	37.9	30.5	23.2	16.6	32.5	26.4	20.4
Some College & Associate	33.2	38.8	18.0	25.0	30.8	37.6	16.8	23.1	30.0	38.2	19.0	27.4
Bachelor's Degree	22.3	9.4	6.4	18.6	27.1	7.0	5.9	15.1	27.4	11.2	6.2	22.7
Professional or Graduate Degree	12.0	4.8	2.8	11.5	16.0	3.2	2.1	10.1	18.7	6.8	2.5	18.1
**Primary Payer**												
Unknown	2.9	2.5	0.1	0.9	2.4	2.0	0.0	0.7	2.2	2.1	0.4	0.6
Private	47.3	28.7	15.3	34.6	52.3	23.2	8.6	26.5	50.6	25.2	7.0	36.4
Medicaid	35.2	56.7	73.8	46.8	27.7	61.7	72.7	54.2	25.8	54.1	74.9	38.8
Other Public	12.1	10.7	5.3	11.4	16.8	12.6	12.7	15.1	20.3	18.4	11.0	22.7
Uninsured	2.4	1.4	5.5	6.3	0.8	0.4	6.0	3.6	1.0	0.4	6.7	1.6
**Prepregnancy BMI**												
Unknown	3.3	2.8	1.6	3.6	3.5	2.7	1.7	3.3	3.0	2.7	1.8	3.9
Underweight	1.1	1.1	5.9	1.6	1.2	1.4	1.3	1.6	0.7	0.5	0.3	0.6
Normal	40.1	26.3	31.0	41.1	46.2	25.7	36.8	41.0	39.9	25.1	31.9	40.9
Overweight	25.4	24.3	30.9	27.4	24.1	23.6	30.9	29.0	26.2	24.1	29.1	27.3
Obesity	30.1	45.5	30.7	26.4	25.0	46.5	29.4	25.1	30.3	47.6	36.9	27.3
**WIC Program**												
Yes	24.7	51.6	38.5	29.0	25.3	62.0	44.5	34.8	24.2	58.3	43.0	32.2
No	73.9	46.7	60.3	68.4	73.4	36.3	53.5	62.4	74.8	40.7	56.7	65.7

© 2022 Hung P et al. *JAMA Network Open.*

Unknown	1.4	1.6	1.2	2.6	1.3	1.7	2.0	2.8	1.0	1.0	0.3	2.1
**Smoking During Pregnancy**	10.6	6.2	0.7	2.6	8.9	5.2	0.8	2.8	6.5	2.5	0.3	1.4
**Previous Cesarean Delivery**	16.1	18.8	17.8	16.3	14.7	17.9	18.9	15.3	16.1	17.7	17.4	15.5
**Preexisting Diabetes**	0.9	1.8	1.5	1.4	0.9	2.2	0.7	1.2	1.0	1.7	1.8	1.0
**Gestational Diabetes**	7.1	6.6	10.2	9.3	6.7	5.8	6.6	7.4	5.9	5.9	6.0	7.2
**Preexisting Hypertension**	2.9	6.9	2.5	2.1	2.7	6.3	0.8	1.7	2.3	6.1	2.2	2.4
**Pregnancy-induced Hypertension**	9.6	11.8	10.8	6.5	7.3	8.9	3.2	5.4	6.5	7.2	4.7	4.8
**Preterm Labor**	9.1	13.6	9.1	9.4	8.4	13.9	6.5	9.6	8.1	12.6	8.1	11.6
**Plurality**												
Singleton	98.3	97.8	98.8	98.7	98.4	97.9	99.1	98.6	98.5	98.0	98.6	97.9
Multiple	1.7	2.2	1.2	1.3	1.6	2.1	0.9	1.4	1.5	2.0	1.4	2.1
**Urban/Rural Location of Facility**												
Unknown	2.9	2.5	0.0	0.9	2.3	2.0	0.0	0.6	2.2	2.0	0.0	0.6
Rural	7.1	6.4	8.6	5.8	30.0	33.3	66.3	31.0	13.7	18.3	11.0	4.8
Urban	90.0	91.1	91.5	93.3	67.7	64.7	33.7	68.4	84.1	79.7	89.0	94.6

Notes: WIC = Women, Infant, Children. NH = Non-Hispanic. Residential segregation measure (isolation index) represents the probability that Black residents would interact with White residents and vice versa across census tracts in a maternal residential county, categorized into low (<40), medium (40-59), and high (>60).

© 2022 Hung P et al. *JAMA Network Open.*

eTable 3. The COVID-19 Pandemic Changes in Severe Maternal Morbidity and Its Racial and Ethnic Disparities by Racial Residential Segregation

		Severe Maternal Morbidity with Blood Transfusion		Severe Maternal Morbidity without Blood Transfusion	
		**Odds Ratios (95 Confidence Interval)**[a]	P Value	**Odds Ratios (95 Confidence Interval)**[a]	P Value
**The COVID-19 Pandemic Difference-in-Difference**					
Pre-pandemic Monthly Trend		**0.98 (0.97, 1.00)**	**0.013**	1.00 (0.99, 1.01)	0.932
Change in Level After March 2020		1.19 (0.85, 1.67)	0.315	0.90 (0.66, 1.23)	0.512
Change in Monthly Trend Peri- vs. Pre-Pandemic		**1.05 (1.02, 1.08)**	**0.005**	**1.02 (1.01, 1.05)**	**0.031**
**Maternal Race/Ethnicity Disparities by Segregation**					
Low Segregation	Black vs. White	**1.47 (1.11, 1.96)**	**0.007**	1.12 (0.94, 1.33)	0.201
	Hispanic vs. White	**0.48 (0.25, 0.91)**	**0.025**	**0.55 (0.39, 0.76)**	**<.001**
	Other Race vs. White	1.41 (0.91, 2.20)	0.124	1.00 (0.63, 1.59)	0.992
Medium Segregation	Black vs. White	**1.52 (1.07, 2.17)**	**0.020**	1.18 (0.95, 1.46)	0.143
	Hispanic vs. White	1.30 (0.45, 3.73)	0.623	**0.56 (0.41, 0.77)**	**<.001**
	Other Race vs. White	**2.13 (1.16, 3.89)**	**0.014**	1.07 (0.68, 1.70)	0.767
High Segregation	Black vs. White	**2.12 (1.38, 3.26)**	**0.001**	1.21 (0.87, 1.66)	0.252
	Hispanic vs. White	**1.90 (1.07, 4.17)**	**0.018**	0.75 (0.48, 1.19)	0.228
	Other Race vs. White	1.66 (0.80, 3.46)	0.174	**1.49 (1.05, 2.12)**	**0.025**
**Maternal Age**					
<25		reference	-	reference	-
25-34		1.11 (0.89, 1.39)	0.365	**1.28 (1.07, 1.54)**	**0.007**
35+		**1.64 (1.20, 2.24)**	**0.002**	**1.74 (1.37, 2.20)**	**<.001**
**Maternal Education**					
No High School Diploma		reference	-	reference	-
High School Diploma		0.88 (0.67, 1.16)	0.360	0.91 (0.75, 1.11)	0.339
Some College		0.85 (0.63, 1.13)	0.258	0.86 (0.68, 1.10)	0.230
Bachelor's Degree		**0.49 (0.35, 0.68)**	**<.001**	**0.67 (0.54, 0.84)**	**0.001**
Graduate School		**0.54 (0.35, 0.83)**	**0.005**	0.75 (0.55, 1.03)	0.074
**Primary Payer**					
Private		reference	-	reference	-
Medicaid		**1.47 (1.17, 1.84)**	**0.001**	1.15 (0.97, 1.35)	0.108
Other Public		**1.41 (1.07, 1.86)**	**0.014**	1.14 (0.91, 1.44)	0.254
Uninsured		1.67 (0.92, 3.02)	0.091	1.00 (0.60, 1.68)	0.996
**Prepregnancy BMI**					
Underweight		1.16 (0.57, 2.37)	0.684	**1.77 (1.16, 2.70)**	**0.008**
Normal		reference	-	reference	-
Overweight		0.92 (0.74, 1.14)	0.441	0.99 (0.85, 1.16)	0.899
Obesity		**0.75 (0.61, 0.93)**	**0.007**	0.94 (0.79, 1.14)	0.545
**WIC Program Enrollment**		0.87 (0.72, 1.05)	0.15	1.03 (0.75, 1.42)	0.857
**Prenatal Care Initiation**					
First Trimester		reference	-	reference	-
After First Trimester		1.04 (0.86, 1.26)	0.660	1.09 (0.93, 1.28)	0.300
**Smoking during Pregnancy**					
Yes		1.23 (0.88, 1.72)	0.234	1.10 (0.84, 1.43)	0.492
No		reference	-	reference	-
**Previous Cesarean**		**2.96 (2.34, 3.74)**	**<.001**	**1.74 (1.43, 2.13)**	**<.001**

© 2022 Hung P et al. *JAMA Network Open.*

**Preexisting/Gestational Diabetes**	0.83 (0.61, 1.13)	0.228	1.01 (0.78, 1.31)	0.939
**Preexisting/Pregnancy-induced Hypertension**	**2.81 (2.24, 3.52)**	**<.001**	**2.02 (1.74, 2.33)**	**<.001**
**Preterm Labor**	**8.53 (6.82, 10.68)**	**<.001**	**4.59 (3.92, 5.39)**	**<.001**
**Plurality**		<.001		0.976
Singleton	reference		reference	
Multiple	4.39 (2.62, 7.37)		1.01 (0.68, 1.48)	
**Urban/Rural Location of Facility**				
Rural	**2.26 (1.75, 2.92)**	**<.001**	1.24 (0.87, 1.76)	0.236
Urban	reference		reference	
**Level of Perinatal Care**				
Level I	reference		reference	
Level II	**4.00 (2.75, 5.81)**	**<.001**	1.17 (0.80, 1.70)	0.431
Level III	**3.26 (2.21, 4.79)**	**<.001**	**2.64 (1.81, 3.85)**	**<.001**
**Parity**				
First Childbirth	reference		reference	
Second Childbirth	**0.53 (0.43, 0.67)**	**<.001**	**0.74 (0.62, 0.88)**	**<.001**
Third or Higher	**0.64 (0.50, 0.82)**	**<.001**	**0.72 (0.60, 0.87)**	**<.001**

Notes: Significant estimates are bold at p<.05; a. models adjusted for county random effects and 95% Confidence Intervals were calculated using modified sandwich variance estimator **with county-level clustering effects.**

© 2022 Hung P et al. *JAMA Network Open.*

eFigure. Monthly and Six-Month Moving Average of Rates of Severe Maternal Morbidity Without Blood Transfusion Among All, White, Black and Hispanic/Latino Women Giving Birth in January 2018-June 2021 by County-Level Black-White Residential Segregation Level

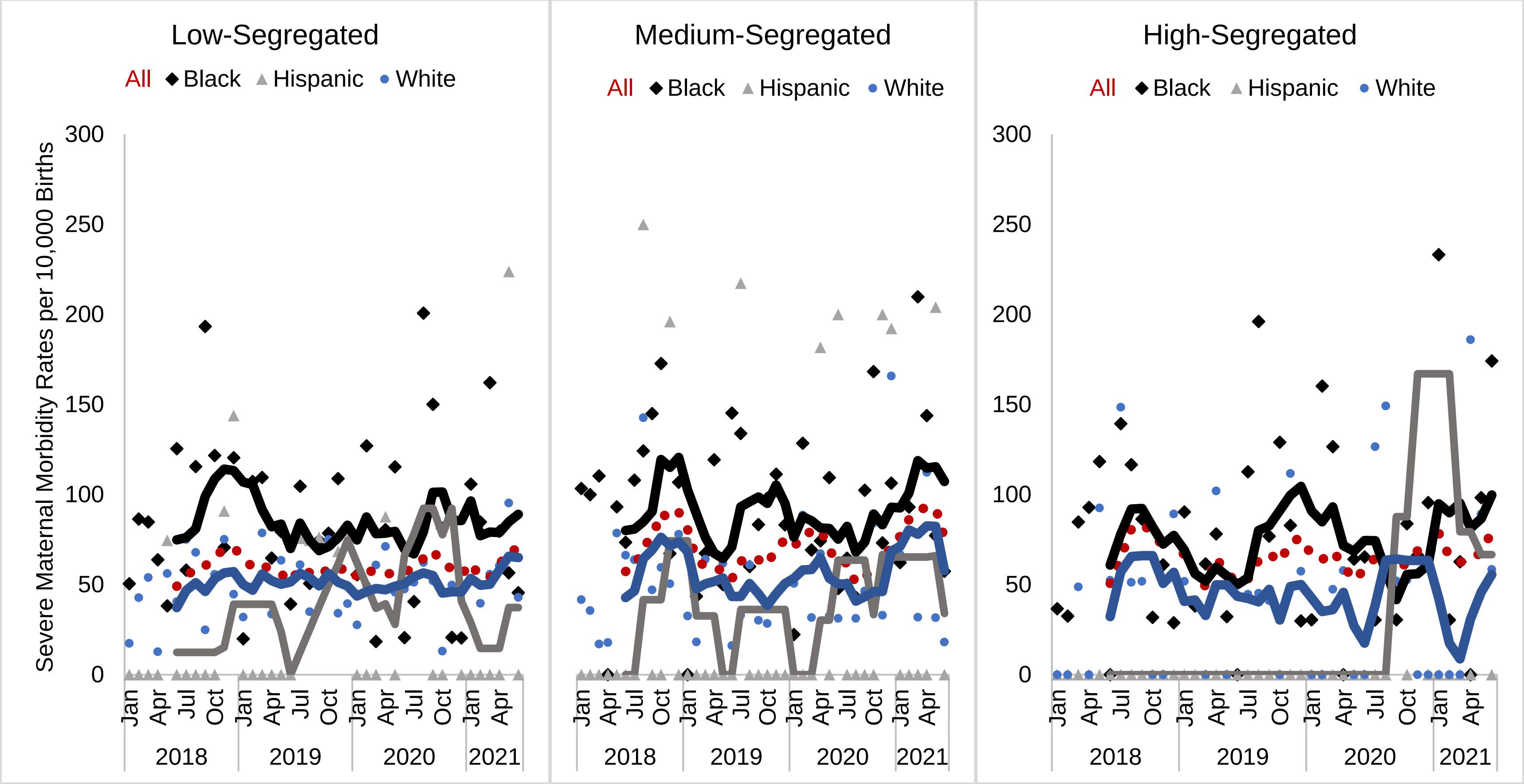

Notes: Residential segregation measure (isolation index) represents the probability that Black residents would interact with White residents and vice versa across census tracts in a maternal residential county, categorized into low (<40%), medium (40%-59%), and high (≥60%). Data for non-Hispanic other race group and some data for Hispanic group are suppressed due to less than 10 cases. All race and ethnicity groups' rates were calculated across all childbirths of non-Hispanic White (White), non-Hispanic Black (Black), Hispanic, and other race populations.

© 2022 Hung P et al. *JAMA Network Open.*
